# Supplementary material for: Effective and Efficient Delivery of Genome-Based Testing-What Conditions Are Necessary for Health System Readiness?
Source: Healthcare (Basel). 2022 Oct 19;10(10):2086. doi: 10.3390/healthcare10102086 (PMC9602865; doi:10.3390/healthcare10102086)
Supplement: Supplementary file 1 [file healthcare-10-02086-s001.zip › healthcare-1903621-supplementary.pdf]

Lab Organization Search
February 25, 2022

Database(s): **Ovid MEDLINE(R) ALL** 1946 to February 25, 2022
Search Strategy:

#	Searches	Results
1	*diagnostic services/ or *clinical laboratory services/ or *genetic testing/ or *Molecular Diagnostic Techniques/	29363
2	exp *"Organization and Administration"/	768370
3	*"delivery of health care"/ or *"delivery of health care, integrated"/	74744
4	2 or 3	829847
5	1 and 4	1436
6	((laboratory or laboratories or ((genetic or molecular) adj (testing or diagnostic*))) adj2 (best practice* or plan* or service* or manag* or infrastructure or (deliver* adj2 (care or service*)) or organiz* or organis* or integrat* or administ* or harmonis* or harmoniz* or structur* or restructur*)).ti.	1715
7	5 or 6	3131
8	Diagnostic Services/og, st [Organization & Administration, Standards]	537
9	Clinical Laboratory Services/og, st [Organization & Administration, Standards]	464
10	*Genetic Testing/og, st [Organization & Administration, Standards]	1035
11	*Laboratories/og, sd	1703
12	or/8-11	3720
13	7 or 12	6426
14	(Animals/ or Models, Animal/ or Disease Models, Animal/) not Humans/	4930268
15	((animal or animals or canine* or dog or dogs or feline or hamster* or lamb or lambs or mice or monkey or monkeys or mouse or murine or pig or pigs or piglet* or porcine or primate* or rabbit* or rats or rat or rodent* or sheep* or veterinar*) not (human* or patient*)).ti,kf,jw.	2447417
16	14 or 15	5361308
17	13 not 16	6205
18	limit 17 to yr="2012 -Current"	2278

Google advanced Scholar and Google Advanced:
laboratory or lab organization OR integration OR planning OR services OR trends OR infrastructure "genetics" filetype:pdf
